# Adult-onset Alexander disease, associated with a mutation in an alternative *GFAP* transcript, may be phenotypically modulated by a non-neutral *HDAC6* variant

**DOI:** 10.1186/1750-1172-8-66

**Published:** 2013-05-01

**Authors:** Laura Melchionda, Mingyan Fang, Hairong Wang, Valeria Fugnanesi, Michela Morbin, Xuanzhu Liu, Wenyan Li, Isabella Ceccherini, Laura Farina, Mario Savoiardo, Pio D’Adamo, Jianguo Zhang, Alfredo Costa, Sabrina Ravaglia, Daniele Ghezzi, Massimo Zeviani

**Affiliations:** 1 Unit of Molecular Neurogenetics, Fondazione Istituto Neurologico ‘Carlo Besta’, Istituto di Ricovero e Cura a Carattere Scientifico (IRCCS), Milan, Italy; 2 BGI-Shenzhen, Shenzhen, China; 3 Unit of Neuropathology and Neurology 5, Fondazione Istituto Neurologico ‘Carlo Besta’, IRCCS, Milan, Italy; 4 BGI-Europe, Copenhagen, Denmark; 5 Laboratory of Molecular Genetics, G Gaslini Institute, Genoa, Italy; 6 Department of Neuroradiology, Fondazione Istituto Neurologico ‘Carlo Besta’, IRCCS, Milan, Italy; 7 Medical Genetics, IRCCS, Burlo Garofolo, University of Trieste, Trieste, Italy; 8 T-Life Research Center, Fudan University, Shanghai, China; 9 National Institute of Neurology, IRCCS ‘C Mondino’, Pavia, Italy

## Abstract

**Background:**

We studied a family including two half-siblings, sharing the same mother, affected by slowly progressive, adult-onset neurological syndromes. In spite of the diversity of the clinical features, characterized by a mild movement disorder with cognitive impairment in the elder patient, and severe motor-neuron disease (MND) in her half-brother, the brain Magnetic Resonance Imaging (MRI) features were compatible with adult-onset Alexander’s disease (AOAD), suggesting different expression of the same, genetically determined, condition.

**Methods:**

Since mutations in the alpha isoform of glial fibrillary acidic protein, GFAP-α, the only cause so far known of AOAD, were excluded, we applied exome Next Generation Sequencing (NGS) to identify gene variants, which were then functionally validated by molecular characterization of recombinant and patient-derived cells.

**Results:**

Exome-NGS revealed a mutation in a previously neglected GFAP isoform, GFAP-ϵ, which disrupts the GFAP-associated filamentous cytoskeletal meshwork of astrocytoma cells. To shed light on the different clinical features in the two patients, we sought for variants in other genes. The male patient had a mutation, absent in his half-sister, in X-linked histone deacetylase 6, a candidate MND susceptibility gene.

**Conclusions:**

Exome-NGS is an unbiased approach that not only helps identify new disease genes, but may also contribute to elucidate phenotypic expression.

## Background

Alexander’s disease (AD, OMIM #203450) is a rare neurological disorder characterized by a peculiar form of leukodystrophy, with infantile, juvenile and adult forms manifesting with different clinical and pathological signs [1]. AD is a sporadic or autosomal dominant condition associated in most of the cases with heterozygous mutations in the gene encoding the glial fibrillary acidic protein, GFAP, an intermediate filament component of the cytoskeleton of several cell types [2]. GFAP mutations frequently occur *de novo*, particularly in infantile cases, while in Adult-onset AD (AOAD) both *de novo* mutations and autosomal dominant transmission have been described [3]. GFAP-containing eosinophil aggregates, known as Rosenthal fibers, distributed in the white matter of the CNS, constitute the morphological hallmark of the disease [2]. Whilst the infantile form shows extensive white matter lesions and usually fatal outcome, AOAD is characterized by predominant brainstem involvement and survival into adulthood [4].

We here report the results of exome next-generation DNA sequencing (NGS) conducted on a family with two maternal half-siblings, affected by two distinct adult-onset neurological syndromes: mild cognitive deterioration and movement disorder in a female patient, motor-neuron disease (MND) in her half-brother. The two patients shared the same mother, but had different, unrelated fathers, suggesting either an X-linked or an autosomal dominant condition with variable penetrance and expressivity. In spite of the diversity of the clinical features, the brain MRI features were compatible with AOAD. However, standard sequence analysis of the nine canonical exons encoding the predominant isoform, GFAP-α, had previously ruled out mutations in both patients.

NGS is a holistic, unbiased approach that generates comprehensive information on gene variance [5]. Exome NGS analysis in our family revealed a heterozygous missense mutation in an alternative exon of the *GFAP* gene (exon 7A), which has not previously been included in the diagnostic screening of AOAD. Additional variants in other genes included a private mutation in the X-linked gene encoding histone deacetylase 6, *HDAC6*, which was present in the male, but absent in the female, patients. HDAC6 was suggested to have a modulating role in different processes related to neurodegeneration, including authophagy, proteosomal degradation, aggresome formation [6,7]. We demonstrated that the mutant HDAC6 variant has reduced deacetylase activity, which could contribute to the different phenotypes of our patients.

## Patients and methods

### Case reports

**Figure 1:**
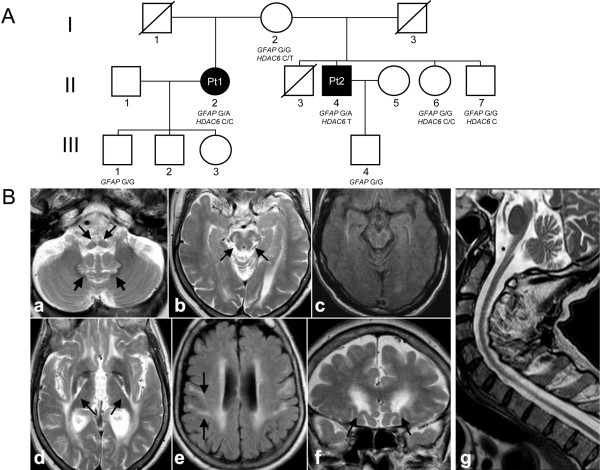
**Pedigree and radiological features of the patients. A**: Pedigree of the family. Black symbols indicate affected patients 1 and 2 (Pt1, Pt2). Genotypes of each tested individual are indicated under the corresponding symbols (GFAP G: wt; GFAP **A**: mutant; HDAC6 C: wt; HDAC6 T: mutant). I-3 died of colon cancer at 62 years of age; I-1died of unknown causes when he was over 80; II-3 died of a cerebral stroke at 60 years of age. **B**: Brain MRI findings of Pt1 (a-f) and Pt2 (g). Atrophy of the medulla is present, with signal abnormalities of the pyramidal tract and medial lemniscus (a, arrows). In the cerebellum, the hylum of the dentate nucleus is bilaterally hyperintense (a, thick arrows). At midbrain level (b,c), substantia nigra and medial lemniscus are hyperintense (b, arrows); a sub-pial rim of high signal intensity is present in the FLAIR image (c). Symmetrical signal abnormalities involve the pallida, particularly at the interface with the posterior limb of the internal capsule (d, arrows). Hyperintensitiy is present in the periventricular white matter, pre- and post-central gyri (e, arrows on the right) and subcortical frontobasal areas (f, arrows). The typical tadpole appearance of the brainstem and cervical spinal cord is seen in the midline sagittal section of Pt2 (g).

Patient 1, Pt1 (subject II-2 in Figure 1A) is now 68 years old. Her insidious disease onset started at 55 years, and was first characterized by psychiatric symptoms, initially as a bipolar disorder with depression alternated by hypomanic behavior (compulsive gambling), and eventually as a cognitive deterioration with apathy, neglect of personal care, and memory loss. Shortly thereafter, she manifested an ataxic gait with frequent falls, followed by progressive dysarthria, dysphagia to liquids, drooling, and fluctuating palatal myoclonus. An Electroencephalography at 61 showed unspecific irritative abnormalities; visual evoked potentials were altered. The neurological examination disclosed a moderate ataxic gait requiring a can, dysarthria, palatal myoclonus, and hypotonia (right > left), increased tendon reflexes, a positive Babinski sign at the right foot, mild dyskinesias, mild distal dystonia. Eye movements were normal. A Mini Mental State Examination scored 16/30. The syndrome slowly progressed, with worsening of cognitive deterioration, dysarthria and dysphagia, and onset of urinary incontinence. Several Electromyography (EMG) examinations have consistently been normal over time.

Pt2 (II-4 in Figure 1A), now 60 years old, was first referred to us at 52, for insidiously progressive walking difficulties, initiated at 46 years with stiffness and weakness at the right lower limb, followed within 3-5 years by involvement of the right upper, and then left lower and upper limbs. He also reported symptoms consistent with nocturnal lower-limb myoclonus. The neurological examination at 52 years showed spastic tetraparesis, more prominent on the right side and lower limbs, bilateral pes equinovarus, normal strength, bilateral Babinski sign. His gait was paraparetic with bilateral thigh adduction; however he could still walk unassisted. He showed no muscle wasting, with the exception of bilateral atrophy of the *temporalis* muscle. He was diagnosed as having “primary lateral sclerosis” and started riluzole and baclofen, with no tangible benefit. Over the subsequent two years he developed mild spastic hypophonia, and moderate dysphagia for liquids, with worsening of the limb spasticity. At 56 he became wheelchair-bound, severely dysphonic and dysphagic, with severe tetra-spasticity, flexed posture, bilateral ankle clonus, bilateral Babinski, bilateral hypotrophy of *temporalis*, *interosseus* and *tibialis anterior* muscles. Sensory examination and neurovegetative tests were normal, as were the eye movements. The EMG showed neurogenic abnormalities, without spontaneous fibrillation. Nerve conduction studies showed motor axonal neuropathy at the lower limbs, whereas the peripheral sensory conduction was normal. Taken together, these findings indicate severe motor-neuron disease (MND) of limb and bulbar districts. Symptoms have slowly progressed over time. The patient has no cognitive deterioration.

The MRI findings of these patients were very similar and consistent with the diagnosis of AOAD (Figure 1B). Atrophy of the medulla oblongata and cervical spinal cord (“tadpole” appearance) and signal abnormalities were present in the brainstem, dentate nuclei and supratentorial periventricular white matter. Additional findings, peculiar to our patients, were mild atrophy of the midbrain with T2 hyperintensity of the substantia nigra and medial lemniscus, pallida, and subcortical white matter in the pre- and post-central gyri and frontobasal areas. Interestingly, Pt1, who had more marked cognitive impairment, had slightly more extensive supratentorial white matter involvement.

To quantitatively express the different clinical features of the two siblings, we used the Kurtzke scale [8] (Additional file 1), that scores several functional systems (motor, cerebellar, brainstem, urinary, visual, and cognitive) usually involved in white matter disease, including leukodystrophies. The scores were obtained 13 years after disease onset for Pt1 and 14 years after onset for Pt2. The global functional impairment, as assessed by the final EDSS score, [9] was 3/10 for Pt1 (able to walk, moderate ataxia and cognitive impairment, not requiring institutionalization) and 8.5/10 for Pt2 (confined to bed but with some residual upper limb function). For Pt2, the source of the severe disability was predominantly due to pyramidal dysfunction: we thus assessed both patients by also using the ALS-Severity scale, [10] which scored 33/40 for pt1 (speech 7, deglutition 6, upper limbs 10, lower limbs 10), and 17/40 for pt2 (speech 3, deglutition 8, upper limbs 4, lower limbs 2). The results of instrumental examinations are reported in Table 1.

**Table 1:** Clinical and instrumental assessments

	**Pt1**	**Pt2**
Current age	68	60
Age at onset	55	46
Disease duration at the time of examinations, years	13	14
Instrumental assessment *		
Cognitive: MMSE score	16/30	30/30
EMG	0	Mild motor axonal neuropathy (1)
MEPs Bulbar/UL/LL	N.A./0/0	1/1/3
SEPs UL/LL	1/1	0/1
BAEPs	NA	NA
VEPs	0	0
Autonomic testing	0	0
Clinical scoring **		
Dysarthria/dysphagia	2	2
Gait abnormalities	1	3
Spasticity	0	3
Axial Ataxia	1	0
Limb dysmetria	2	0
Limb weakness	0	2
Muscle wasting	0	1
Sphincter function	2	0

* For neurophysiological assessments, values are graded as 0 (normal), 1 (abnormality not exceeding 25% of upper/lower normal ranges), 2 (between 25 and 50%), and 3 (beyond 50%).

** For clinical dysfunctions, abnormalities are graded as 0 (normal), 1 (only objective signs), 2 (mild dysfunction, not interfering with activities), 3 (severe dysfunction interfering with walking, feeding, or social interactions).

MMSE: mini–mental state examination; EMG: Electromyography; MEPs: Motor evoked potentials; SEPs: Sensory evoked potentials; UL/LL: upper/lower limbs; BAEPs: Brainstem Auditory Evoked Potentials; VEPs: visual evoked potentials.

### Molecular analyses

Informed consent for participation in this study was obtained from all family members, in agreement with the Declaration of Helsinki and approved by the Ethical Committee of the Fondazione Istituto Neurologico – IRCCS, Milan, Italy.

Genomic DNA was extracted by standard methods from peripheral blood samples (I-2, II-2, II-4, II-6, II-7, III-1, III-3) and from skin fibroblasts (II-2, II-4). Whole-exome and Sanger’s sequencing were performed as described [11]. Total RNA was isolated from fibroblasts (RNeasy kit, Qiagen) and then transcribed to cDNA (Cloned AMV first-strand cDNA synthesis kit, Invitrogen). Quantitative Real-time PCR (QRtPCR) was assayed on an ABI Prism 7000 apparatus (Applied Biosystems). Additional file 2 reports primers and conditions for PCR amplifications of relevant exons of human *GFAP* and *HDAC6* and for QRtPCR of *HDAC6* cDNA.

Additional file 3 reports URLs for biocomputational analysis.

A GFP tagged GFAP cDNA (Origene RG225707) was modified by using Quick-change Site-directed mutagenesis kit (Stratagene) to introduce either the c.1289G > A or the c.1288C > T nucleotide change in the RG225707 clone, using primers listed in Additional file 2.

### Cellular experiments

Cell culture, transient transfections, western-blot analysis, and immunocytochemistry were performed as described, [12-15] using antibodies against α-tubulin (Life Science) and acetylated α-tubulin (Sigma). Patients’ fibroblasts and adult control fibroblasts were grown under the same conditions, and analyzed among culture passages 5 and 8. As a positive control for tubulin acetylation, fibroblasts were pre-incubated with the specific HDAC6 inhibitor Tubacin (0, 0.2 μM and 2.5 μM) (Sigma) for 24 h [16]. Immunohistochemistry was carried out on 2 μm thick sections from pellets of Pt1, Pt2 and control fibroblasts, fixed in glutaraldehyde 2.5% (Electron Microscopy Science - EMS), in 0.05 M PBS pH 7.4, dehydrated in graded acetone, and embedded in Spurr (Epoxy resin, EMS).

Transfection of U251-MG by electroporation was performed in triplicate according to the manufacturer’s protocol (GenePulserII-Biorad), and about 100 cells were analyzed blindly for each experiment (a total of 324 cells for GFP-GFAP-ϵ^wt^ and 285 for GFP-GFAP-ϵ^R430H^ in a first experiment, and 460 cells for either GFP-GFAP-ϵ^wt^ or GFP-GFAP-ϵ^R430C^ in a second experiment).

## Results

Mutational screening ruled out mutations in the *SPG4* and *SPG7* genes in Pt2, due to the presence of spastic tetraparaparesis; in the *HTT* gene in Pt1, due to the subtle onset of symptoms consistent with an affective disorder, together with cognitive dysfunction; and in the *UBQLN2* and *C9orf72* genes, recently associated to ALS/FTD, in both.

**Figure 2:**
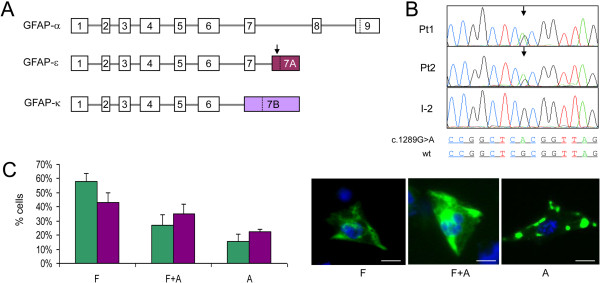
**Characterization of the GFAP c.1289G>A/p.R430H mutation. A**: Schematic representation of the exonic structure of different GFAP isoforms. Dotted lines indicate the termination codons. The arrows indicate the position of the c.1289G>A variant (Note that in GFAP-κ the c.1289G>A mutation is part of the 3′-UTR). **B**: Electropherograms of *GFAP* exon 7A region containing c.1289G>A variant, in patients 1 and 2 (Pt1, Pt2) and in their mother (I-2). **C**: The histogram displays the percentages of cells transfected with GFP-GFAP-ϵ^wt^ (green bars) or GFP-GFAP-ϵ^R430H^ (purple bars), classified in filamentous pattern (F), cytoplasmic aggregates on a filamentous pattern (F + A), cytoplasmic aggregates with no filamentous pattern (A). Scale bars represent 15 μm. A total of 324 cells for GFP-GFAP-ϵ^wt^ and 285 for GFP-GFAP-ϵ^R430H^, from 3 independent experiments, were blindly analyzed by two different operators. ANOVA test for interaction p = 0.001.

The MRI features were consistent with AOAD, but no mutation was detected in the nine exons encoding the prevalent (alpha) isoform of GFAP (GFAP-α, NP_002046.1; Figure 2A). All of the known mutations associated with Alexander’s disease have so far been found in this isoform, [17] which is the only one analyzed by standard screening. However, exome-NGS revealed a heterozygous variant (c.1289G > A, p.R430H) in the alternative *GFAP* exon 7A (Ex7A) in both patients (Figure 2B). Ex7A is part of the transcript encoding the GFAP-ϵ isoform (NP_001124491.1), which differs from GFAP-α in the last 35 amino acids. A third isoform, GFAP-κ (NP_001229305.1), which contains a unique exon 7B, has also been identified (Figure 2A) [18]. The c.1289G>A nucleotide change was absent in the healthy mother and in all other tested family members. DNA samples from I-1 and I-3, fathers of Pt1 and Pt2, respectively, were unavailable. Haplotype analysis of the *GFAP* genomic region by SNPs array in the available family members confirmed that the father of Pt1 was different from that of Pt2 and of his siblings, whilst Pt1 and Pt2 share the same maternal allele (Additional file 4). Since the likelihood that the same rare variant (<0.01%) may occur independently in the two patients is negligible, the most probable hypothesis is that the mutation was transmitted by descent to both Pt1 and Pt2 by maternal germinal mosaicism, a mechanism that can also explain the healthy status of the mother. Since blood was the only source of DNA available from the mother, somatic mosaicism affecting other tissues of this subject cannot be excluded, as recently found in an AD patient with atypical infantile clinical presentation and essentially normal MRI features [19]. However, we think that the latter hypothesis is unlikely, since no trace of mutation could be detected by an *ad hoc* RFLP analysis carried out in the mother’s DNA (not shown) and, in contrast with the case reported by Flint et al. [19], this lady is now 87 years old and well.

In contrast with a p.R430C SNP (rs 78994946), reported with a frequency of 1% in dbSNP, the p.R430H change found in our patients is absent in both dbSNP and the Exome Variant Server (EVS) database, which contains >10000 alleles (≈7000 of European origin). These data are compatible for p.R430H being a deleterious mutation (Additional file 5).

GFAP is an intermediate filament (IF) protein expressed mainly by astrocytes and ependymocytes. Recent data suggested that GFAP-ϵ was unable to form filaments by itself but it could participate to the formation of the GFAP network by interacting with GFAP-α [20]. Hence we analyzed the IF meshwork in human astrocytoma U251-MG cells, constitutively expressing both GFAP-α and GFAP-ϵ, by expressing GFP-tagged wt and mutated GFAP-ϵ (GFP-GFAP-ϵ^wt^ vs. GFP-GFAP-ϵ^R430H^). Cells were assigned to three patterns: [14] (i) exclusively filamentous pattern (F), (ii) cytoplasmic aggregates on a filamentous pattern (F + A), (iii) cytoplasmic aggregates with no filamentous pattern (A). The expression of GFP-GFAP-ϵ^wt^ led to a distribution among the three groups similar to that reported for GFP-GFAP-α^wt^[14] (Figure 2C) indicating no intrinsic damaging effect of recombinant GFP-GFAP-ϵ^wt^ in our experimental conditions. Contrariwise, expression of mutant GFP-GFAP-ϵ^R430H^ produced significant decrease in F (43% vs. 58%; test t p = 0.002) and increase in A (22% vs. 15%; test t p = 0.009) cells (Figure 2C), with a distinct distribution in the three patterns compared to GFP-GFAP-ϵ^wt^ expressing cells (ANOVA test for interaction p = 0.001). Notably, the expression of GFP-tagged GFAP carrying the R430C variant (GFP-GFAP-ϵ^R430C^) led to a distribution amongst the three different patterns similar to that obtained with GFP-GFAP-ϵ^wt^, i.e. non-significant (ANOVA test for interaction p = 0.333). These results indicate that GFAP-ϵ^R430H^ is inefficiently incorporated, and is likely to perturb the GFAP network in GFAP-expressing astrocytoma cells, whereas the GFAP-ϵ^R430C^ variant is functionally wt, but we cannot exclude the possibility that variations in the level of expression contributed to this result.

**Figure 3:**
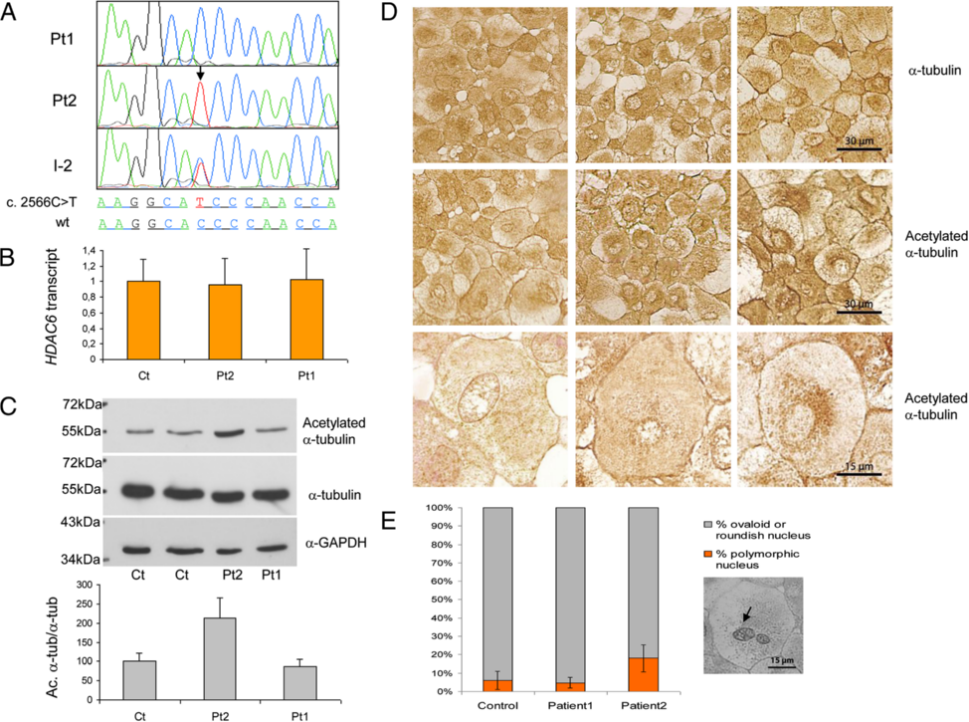
**Characterization of the HDAC6 c.2566C>T/p.P856S variant. A**: Electropherograms of *HDAC6* exon 25 region containing the c.2566C>T variant, in patients 1 and 2 (Pt1, Pt2) and their mother (I-2). **B**: Levels of *HDAC6* transcript, normalized to that of the endogenous *GAPDH* cDNA, in controls (Ct; mean of five subjects) and patients 1 and 2 (Pt1, Pt2), obtained from 3 independent experiments. Two-tailed Student’s t-tests showed no significant differences: Pt2 vs. Ct p = 0.811; Pt2 vs. Pt1 p = 0.813; Pt1 vs. Ct p = 0.896. **C**: Exemplifying Western-blot analysis of fibroblast lysates from control subjects (Ct1, Ct2) and patients 1 and 2 (Pt1, Pt2), using antibodies against acetylated α-tubulin (upper panel), α-tubulin (middle panel) and α-GAPDH, as loading control (lower panel). The graph represents the ratio acetylated α-tubulin/α-tubulin obtained by densitometric analysis from 3 independent experiments: 100% corresponds to the mean value of four control subjects. Two-tailed Student’s t-test Pt2 vs Ct p = 0.002, Pt1 vs Ct p = 0.33. **D**: Immunocytochemistry on fibroblasts from a control subject (Ct) and patients 1 and 2 (Pt1, Pt2), using antibodies against α-tubulin and acetylated α-tubulin. Scale bars are reported on the right for each row. **E**: Percentages of multilobated nuclei in control, Patient1 and Patient2. A total of 15 digital images (at least 600 cells for each patient) representative of the whole sections were collected and analyzed for each sample; the arrow indicates a typical multilobated nucleus. Two-tailed Student’s t-test between Control vs. Pt1 showed no significant differences (p = 0.4970); Pt2 vs. Control p = 0.000099; Pt2 vs. Pt1 p = 0.000018 (both highly significant).

To test whether additional genes could influence phenotype expression, 18 genes with variants in Pt2 were prioritized by the Endeavour software, [21] using “training genes” associated with MND (Additional file 6). The highest score was achieved by *HDAC6*, on chromosome Xp11.23, encoding a member of the histone deacetylase family (NP_006035.2); Pt2 was hemizygous for a c. 2566C>T/p.P856S, variant, whereas Pt1, II-6 and II-7 were wt, and the mother, I-2, was heterozygous (Figure 3A). Whilst the variants in the other genes were all relatively frequent SNPs and/or present also in Pt1 (Additional file 6), the P856S change was absent in all available databases, including EVS. The amount of *HDAC6* transcripts was similar in fibroblasts from Pt2 vs. Pt1 or control subjects, indicating that neither HDAC6 expression nor stability is severly affected by the mutation (Figure 3B). However, acetylated alpha-tubulin, a HDAC6 substrate, [22] was consistently increased (Figure 3C); treatment of fibroblasts with tubacin, a selective HDAC6 inhibitor, clearly increased the acetylation of alpha-tubulin, confirming the specificity of this assay to detect impaired HDAC6 activity (Additional file 7).

Densitometric analysis of immunoreactive bands from three independent experiments, showed that the ratio acetylated α-tubulin/α-tubulin was significantly augmented to 213% in Pt2, compared to the mean value of four control subjects, but was unchanged (87%) in Pt1 (Figure 3C). Moreover, immunocytochemical staining showed abnormal clumps of acetylated α-tubulin in the perinuclear region of Pt2 fibroblasts (Figure 3D). Interestingly HDAC6^P856S^ fibroblasts showed a significantly higher number of multilobated nuclei, compared to control cells, which could be consequent to altered physical connection between nuclear membrane and cytoskeletal network (Figure 3E). Taken together these results suggest dysregulation of the microtubule-organizing center (MTOC), associated with reduced HDAC6 activity [23].

## Discussion

A substantial fraction of AOAD patients are sporadic, the most frequent symptoms being related to bulbar dysfunction, pyramidal involvement and cerebellar ataxia. Palatal myoclonus is frequent in, and highly suggestive of, AOAD [4]. Other findings include cognitive deterioration, sleep disorders, and dysautonomia. The course is slowly progressive and fluctuations may occur. Ultimately, the diagnosis is strongly suggested by a typical MRI pattern, and confirmed by *GFAP* gene analysis. In our family, Pt1 has been suffering of slowly progressive cognitive impairment and mild movement disorder, whereas her younger half-brother (Pt2) has severe MND. In spite of clinical diversity, the cardinal MRI features of AOAD [24] were present in both. The absence of mutation in the GFAP-α encoding gene prompted us to perform exome-NGS and eventually identify a unique mutation in alternative *GFAP* ex7A, not present in the healthy mother tested DNAs and with a deleterious outcome in a cellular model. These are in fact the first cases associated with a mutation in the GFAP-ϵ variant (GFAP-ϵ^R430H^). Whilst this finding supports the idea that AOAD is almost invariably associated with abnormalities of *GFAP*, it also expands the spectrum of variants that should be included in the diagnostic screening. Due to the pedigree structure, the mutation has very likely been transmitted by maternal germinal mosaicism, since it was absent in other available family members, including the healthy mother of the two patients.

The clinical diversity in our two half-siblings was as remarkable as to suggest that differential segregation of other gene variants could influence phenotypic expression. A prioritized variant found by *in*-*silico* data mining was in *HDAC6*. A hemizygous HDAC6^P856S^ change, found in Pt2, and absent in Pt1, was associated with decreased tubulin-specific deacetylase activity [22]. Through deacetylation of α-tubulin, HSP90, and other substrates, and binding to ubiquitinated proteins that are then transported into, and degraded by, the aggresome, HDAC6 plays a role in a number of important homeostatic and signaling pathways, including axonal transport, redox signaling, misfolded-protein response, and autophagy [25,26]. Interestingly, the RNA-binding modulator factors TDP-43 and FUS/TLS, whose mutations are associated with familial amyotrophic lateral sclerosis (ALS), have *HDAC6* mRNA as a specific substrate [27]. A *Drosophila* model in which TDP-43 is silenced shows decreased *HDAC6* expression, [28] and *HDAC6* overexpression is able to rescue the phenotype of a *Drosophila* model of spinobulbar muscular atrophy [6].

Taken together, these observations indicate HDAC6 as a master regulator of different neuroprotective mechanisms, partly mediated by controlling MTOC biogenesis and function, [23] and predict a role for defective HDAC6 in neurodegeneration, particularly in MND [26]. As for mammalian models, although a first strain of *HDAC6* knockout (KO) mice presented no sign of neurodegeneration, [29] altered emotional behaviors suggested a contribution of HDAC6 to maintain proper neuronal activity [30]. Moreover, a second KO *HDAC6* strain displayed ubiquitin-positive aggregates and increased apoptosis of brain nerve cells, both hallmarks of neurodegeneration, starting from 6 months of age [31]. These and other results suggest for HDAC6 a complex role in contributing to either neuroprotection or neurodegeneration, depending on the specific pathological condition [7,26,32]. These opposite effects can indeed hamper the development of therapeutic strategies based on HDAC6 modulation [7].

Albeit preliminary, our own results support the interesting hypothesis that the HDAC6^P856S^ protein variant may be acting synergistically with the GFAP-ϵ^R430H^ mutation, conditioning the development of the severe MND phenotype of Pt2.

The mechanisms underlying the diverse etiology and expressivity of many inherited neurodegenerative disorders are still poorly understood. Exome-NGS is an unbiased approach that not only helps identify new disease genes, but may also contribute to elucidate phenotypic expression and penetrance.

## Competing interests

The authors declare that they have no competing interests.

## Authors’ contributions

LM performed genetic screening and protein characterization. HW, XL, WL were involved in exome-sequencing and bio-informatic analysis, under the supervision of FM and JZ. VF and MM analyzed the morphology of mutant fibroblasts. IC supplied U251 cells and suggestions for transfection studies. LF and MS evaluated the MRI. PDA performed haplotypes analysis. AC, SR and MZ evaluated the patients and wrote the case report. DG monitored genetic/protein analyses, prioritized Pt2 variants, and drafted the paper. MZ supervised all the study, drafted and revised the paper. All authors read and approved the final manuscript. LM and MF share first authorship.

## Supplementary Material

Additional file 1 Clinical involvement on specific functional systems. Click here for file

Additional file 2 Table with primers sequences and amplification conditions. Click here for file

Additional file 3 Table with URLs for biocomputational analysis. Click here for file

Additional file 4 **Haplotype analysis of the *GFAP* genomic region by SNPs array (ILLUMINA HumanCytoSNP-12 BeadChip). Individuals are numbered according to the pedigree in Figure **1A. Click here for file

Additional file 5 Table with predictions of pathogenicity for GFAP p.R430H change. Click here for file

Additional file 6 Tables with Endeavour prioritization and the list of genes with homozygous, compound heterozygous or X-linked variants in patient 2. Click here for file

Additional file 7 Western-blot analysis of control fibroblasts treated with tubacin, a specific inhibitor of HDAC6. Click here for file
