# Supplementary material for: Adult-onset Alexander disease, associated with a mutation in an alternative GFAP transcript, may be phenotypically modulated by a non-neutral HDAC6 variant
Source: Orphanet J Rare Dis. 2013 May 1;8:66. doi: 10.1186/1750-1172-8-66 (PMC3654953; doi:10.1186/1750-1172-8-66)
Supplement: Additional file 1 — Clinical involvement on specific functional systems. [file 1750-1172-8-66-S1.doc]

**Additional file 1**

Clinical involvement on specific functional systems. The bars represent the score on the Kurtzke scale of the two patients (Pt1 red bars; Pt2 blue bars), 13 and 14 years after the onset of the disease(higher scores express higher disability).

* Brainstem involvement consisted of spastic dysarthria and fluids dysphagia for both patients.

P: Pyramidal ; C Cerebellar; B Brainstem; S Sensory; U Urinary; V Visual; Co Cognitive
