# Supplementary material for: Adult-onset Alexander disease, associated with a mutation in an alternative GFAP transcript, may be phenotypically modulated by a non-neutral HDAC6 variant
Source: Orphanet J Rare Dis. 2013 May 1;8:66. doi: 10.1186/1750-1172-8-66 (PMC3654953; doi:10.1186/1750-1172-8-66)
Supplement: Additional file 2 — Table with primers sequences and amplification conditions. [file 1750-1172-8-66-S2.doc]

**Additional file 2**

**Primers sequences and amplification conditions**

| *Amplicon* | *Forward primer* | *Reverse primer* | *PCR conditions* |
| --- | --- | --- | --- |
| *GFAP* exon 7A | AGATCCCTGAGCAAGCACTG | CTGGGAAGAGGGAACTCAGG | Ta: 58°C; GoTaq Promega |
| *GFAP* exon 7B | CCCTCTCCCTCTGCTTTCTT | CGGCGTTCCATTTACAATCT | Ta: 58°C; GoTaq Promega |
| *HDAC6* exon 25 | GGGAACCCAGGGAAGGAG | GAGTGAGGGCCACCACAG | Ta: 58°C; GoTaq Promega |
| *HDAC6* cDNA (nt556-616) | TCGCTGCGTGTCCTTTCAG | GCTGTGAACCAACATCAGCTCTT | Quantitative PCR (ABI Prism7000) |
| *HDAC6* cDNA (nt3605-3675) | TGGGTGTGTCTCTCTTGCTATCA | CCATGGTGTTGGAGCATGTG | Quantitative PCR (ABI Prism7000) |
| *Mutagenesis* |  |  |  |
| *GFAP* c.1288C>T | GAACGCCGCCGGCTTGCGGTACGCGTACGC | GCGTACGCGTACCGCAAGCCGGCGGCGTTC | Quick-change Site-directed mutagenesis kit (Stratagene) |
| *GFAP* c.1289G>A | GAACGCCGCCGGCTCACGGTACGCGTACGC | GCGTACGCGTACCGTGAGCCGGCGGCGTTC | Quick-change Site-directed mutagenesis kit (Stratagene) |

Ta: Annealing temperature
