# Supplementary material for: Adult-onset Alexander disease, associated with a mutation in an alternative GFAP transcript, may be phenotypically modulated by a non-neutral HDAC6 variant
Source: Orphanet J Rare Dis. 2013 May 1;8:66. doi: 10.1186/1750-1172-8-66 (PMC3654953; doi:10.1186/1750-1172-8-66)
Supplement: Additional file 3 — Table with URLs for biocomputational analysis. [file 1750-1172-8-66-S3.doc]

**Additional file 3**

**URLs for biocomputational analysis**

| *Prediction softwares for mutation pathogenicity* | |
| --- | --- |
| MutPred | http://mutpred.mutdb.org |
| PMUT | http://mmb.pcb.ub.es/PMut |
| Polyphen2 | http://genetics.bwh.harvard.edu/pph2 |
| Sorting Intolerant From Tolerant (SIFT) | http://sift.bii.a-star.edu.sg |
| *Public databases for SNPs* | |
| dbSNP | http://www.ncbi.nlm.nih.gov/projects/SNP |
| Exome Variant Server (EVS) | http://evs.gs.washington.edu/EVS |
| HapMap | http://hapmap.ncbi.nlm.nih.gov/ |
| *Computational prioritization of candidates genes* | |
| Endeavour | http://homes.esat.kuleuven.be/~bioiuser/endeavour/index.php |
