# Supplementary material for: Adult-onset Alexander disease, associated with a mutation in an alternative GFAP transcript, may be phenotypically modulated by a non-neutral HDAC6 variant
Source: Orphanet J Rare Dis. 2013 May 1;8:66. doi: 10.1186/1750-1172-8-66 (PMC3654953; doi:10.1186/1750-1172-8-66)
Supplement: Additional file 4 — Haplotype analysis of the GFAP genomic region by SNPs array (ILLUMINA HumanCytoSNP-12 BeadChip). Individuals are numbered according to the pedigree in Figure 1A. [file 1750-1172-8-66-S4.doc]

**Additional file 4**


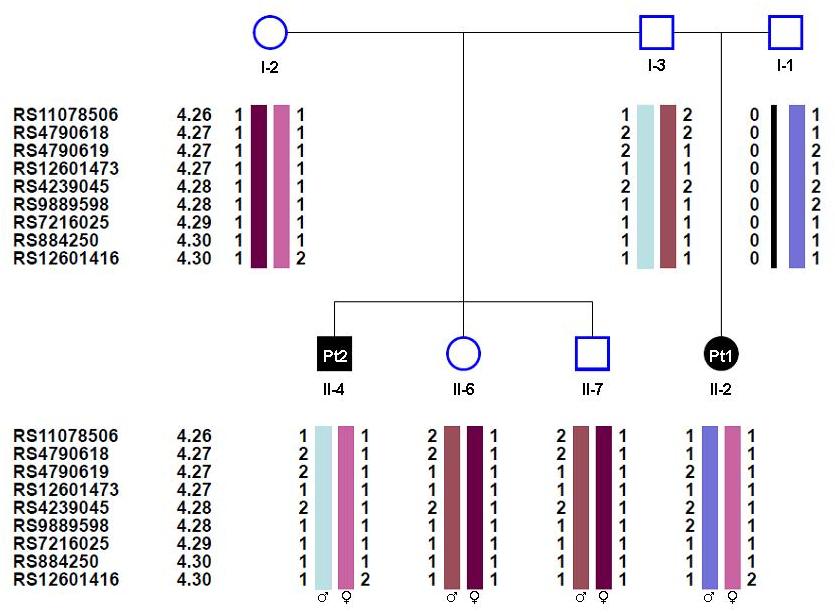


Haplotype analysis of the *GFAP* genomic region by SNPs array (ILLUMINA HumanCytoSNP-12 BeadChip). In the siblings the symbol ♂ indicates the paternal allele and ♀ the maternal allele. Individuals are numbered according to the pedigree in Figure 1A.
