# Supplementary material for: Adult-onset Alexander disease, associated with a mutation in an alternative GFAP transcript, may be phenotypically modulated by a non-neutral HDAC6 variant
Source: Orphanet J Rare Dis. 2013 May 1;8:66. doi: 10.1186/1750-1172-8-66 (PMC3654953; doi:10.1186/1750-1172-8-66)
Supplement: Additional file 5 — Table with predictions of pathogenicity for GFAP p.R430H change. [file 1750-1172-8-66-S5.doc]

**Additional file 5**

**Predictions of pathogenicity for GFAP p.R430H change**

| *Software* | *Prediction result* |
| --- | --- |
| MutPred | Probability of deleterious mutation: 0.240 |
| PMUT | Pathological |
| Polyphen2 | Probability of deleterious mutation: 0.564;  Possibly damaging |
| Sorting Intolerant From Tolerant (SIFT) | Probability that the amino acid change is tolerated: 0.01 |
