# Supplementary material for: Adult-onset Alexander disease, associated with a mutation in an alternative GFAP transcript, may be phenotypically modulated by a non-neutral HDAC6 variant
Source: Orphanet J Rare Dis. 2013 May 1;8:66. doi: 10.1186/1750-1172-8-66 (PMC3654953; doi:10.1186/1750-1172-8-66)
Supplement: Additional file 6 — Tables with Endeavour prioritization and the list of genes with homozygous, compound heterozygous or X-linked variants in patient 2. [file 1750-1172-8-66-S6.doc]

**Additional file 6**

Endeavour prioritization

| **Global prioritization** | **Annotation EnsemblEst** | **Annotation GeneOntology** | **Annotation Interpro** | **Annotation Kegg** | **Expression SonEtAl** | **Expression SuEtAl** | **Motif** | **Text** |
| --- | --- | --- | --- | --- | --- | --- | --- | --- |
| HDAC6 | HDAC6 | HDAC6 | HDAC6 | ABCD1 | HDAC6 | IRAK1 | BDP1 | HDAC6 |
| ENSG00000131721 | BIRC6 | ABCD1 | ABCD1 | PRSS1 | PRSS1 | AFF1 | HDAC6 | ABCD1 |
| VCX3B | AFF1 | AFF1 | AFF1 | IRAK1 | IRAK1 | ENSG00000196604 | VCX3B | HRNR|RPTN |
| AFF1 | SDK1 | GZMH | GZMH | ~~HDAC6~~ | GZMH | GZMH | ENSG00000131721 | BIRC6 |
| BDP1 | GZMH | BDP1 | BDP1 | ~~AFF1~~ | CCDC120 | HDAC6 | ABCD1 | CCDC63 |
| ABCD1 | HRNR|RPTN | CCDC39 | ENSG00000131721 | ~~GZMH~~ | SDK1 | VCX3B | CXorf55 | CCDC120 |
| CCDC120 | BDP1 | ENSG00000131721 | BIRC6 | ~~BDP1~~ | ADAMTS3 | ABCD1 | ENSG00000196604 | AFF1 |
| CXorf55 | ABCD1 | VCX3B | SDK1 | ~~ENSG00000131721~~ | BDP1 | ADAMTS3 | AFF1 | VCX3B |
| HRNR|RPTN | ADAMTS3 | BIRC6 | HRNR|RPTN | ~~BIRC6~~ | BIRC6 | PRSS1 | ADAMTS3 | ENSG00000131721 |
| ENSG00000196604 | PRSS1 | SDK1 | ADAMTS3 | ~~SDK1~~ | ABCD1 | ~~CCDC120~~ | CCDC63 | SDK1 |
| CCDC63 | CXorf55 | HRNR|RPTN | PRSS1 | ~~HRNR|RPTN~~ | AFF1 | ~~SDK1~~ | PRSS1 | PRSS1 |
| GZMH | CCDC39 | ADAMTS3 | ENSG00000196604 | ~~ADAMTS3~~ | ~~ENSG00000131721~~ | ~~BDP1~~ | SDK1 | BDP1 |
| SDK1 | ENSG00000196604 | PRSS1 | IRAK1 | ~~ENSG00000196604~~ | ~~HRNR|RPTN~~ | ~~BIRC6~~ | HRNR|RPTN | ADAMTS3 |
| IRAK1 | CCDC63 | ENSG00000196604 | CCDC120 | ~~CCDC120~~ | ~~ENSG00000196604~~ | ~~ENSG00000131721~~ | CCDC120 | GZMH |
| CCDC39 | ~~ENSG00000131721~~ | IRAK1 | ~~CCDC39~~ | ~~CCDC39~~ | ~~CCDC39~~ | ~~HRNR|RPTN~~ | CCDC39 | IRAK1 |
| BIRC6 | ~~CCDC120~~ | ~~CXorf55~~ | ~~VCX3B~~ | ~~VCX3B~~ | ~~VCX3B~~ | ~~CCDC39~~ | GZMH | ~~CXorf55~~ |
| ADAMTS3 | ~~IRAK1~~ | ~~CCDC63~~ | ~~CXorf55~~ | ~~CXorf55~~ | ~~CXorf55~~ | ~~CXorf55~~ | BIRC6 | ~~ENSG00000196604~~ |
| PRSS1 | ~~VCX3B~~ | ~~CCDC120~~ | ~~CCDC63~~ | ~~CCDC63~~ | ~~CCDC63~~ | ~~CCDC63~~ | ~~IRAK1~~ | ~~CCDC39~~ |

### Legend

**Note:** The top 16 genes are attributed a color in order to easily find the rank of a given gene obtained for each model (selected data source).

ELN Candidates ranking in the top 16 are attributed a random background color.

ELN Candidates with global rank > 16 have a white background color.

ELN Candidates in red color achieved a maximum dissimilarity score (most dissimilar to the training genes).

~~ELN~~ Candidates displayed in a line through font were not scored for the data source (data missing).

**List of the genes with homozygous, compound heterozygous or X-linked variants in patient 2**

| **Chromosome** | **Position** | **Reference** | **GeneName** | **Pt2** | **Pt1** | **notes /EVS %** | **Codons** | **aa change** |
| --- | --- | --- | --- | --- | --- | --- | --- | --- |
| chr1 | 152187657 | C | HRNR | G/A | G/A |  | GGA6448AGA | G2150R |
| chr1 | 152187606 | G | HRNR | C/T | C/T |  | CGT6499TGT | R2167C |
| chr2 | 32689842 | C | BIRC6 | C/T | wt | 0,9% | CCA5207CTA | P1736L |
| chr2 | 32819010 | G | BIRC6 | G/A | wt | 1,0% | GTA13384ATA | V4462I |
| chr2 | 130877584 | G | POTEF | A | wt | rs201936889 | CAG505AAG | Q169K |
| chr3 | 180359874 | G | CCDC39 | C/T | C/T |  | ACA1781ATA | T594I |
| chr3 | 180366086 | T | CCDC39 | A/G | A/G |  | CAG1229CGG | Q410R |
| chr4 | 73149268 | G | ADAMTS3 | C/G | C/G |  | ACT3203AGT | T1068S |
| chr4 | 73178175 | T | ADAMTS3 | A/G | wt | 1,1% | AAT1754AGT | N585S |
| chr4 | 88035586 | C | AFF1 | C/T | C/T |  | GCG1580GTG | A527V |
| chr4 | 88052985 | C | AFF1 | C/T | C/T |  | CCA3121TCA | P1041S |
| chr5 | 70837349 | G | BDP1 | G/A | wt | *Variants on the same allele* | GTA6091ATA | V2031I |
| chr5 | 70837354 | T | BDP1 | T/A | wt |  | AAT6096AAA | N2032K |
| chr7 | 142459667 | G | PRSS1 | G/A | G/A |  | spliceSite | - |
| chr7 | 142459679 | G | PRSS1 | G/A | G/A |  | spliceSite | - |
| chr7 | 4002309 | C | SDK1 | C/G | wt | 0,8% | CAG1255GAG | Q419E |
| chr7 | 4002375 | G | SDK1 | G/A | wt | 0,9% | GGA1321AGA | G441R |
| chr12 | 111345219 | G | CCDC63 | T | T |  | CGC1631CTC | R544L |
| chr14 | 25076906 | C | GZMH | G/C | G/A |  | CGG251CCG | R84P |
| chr14 | 25076877 | G | GZMH | C/G | C/G |  | CCC280GCC | P94A |
| chrX | 153008476 | T | ABCD1 | C | T/C | *Pseudogene* | TCG1816CCG | S606P |
| chrX | 48925140 | G | CCDC120 | T | G | *Unknown function* | TGG1385TTG | W462L |
| **chrX** | **48681375** | **C** | **HDAC6** | **T** | **C** | **0%** | **CCC2566TCC** | **P856S** |
| chrX | 153284723 | T | IRAK1 | G | T | 0,2% | ATC361CTC | I121L |
| chrX | 114425923 | C | RBMXL3 | A | C | 15,0% | GCC1919GAC | A640D |
| chrX | 119297530 | A | RHOXF2 | G | A | 2,0% | GAC704GGC | D235G |
| chrX | 8433535 | A | VCX3B | C | A/C | 3,0% | AAG44ACG | K15T |
