# Supplementary material for: Adult-onset Alexander disease, associated with a mutation in an alternative GFAP transcript, may be phenotypically modulated by a non-neutral HDAC6 variant
Source: Orphanet J Rare Dis. 2013 May 1;8:66. doi: 10.1186/1750-1172-8-66 (PMC3654953; doi:10.1186/1750-1172-8-66)
Supplement: Additional file 7 — Western-blot analysis of control fibroblasts treated with tubacin, a specific inhibitor of HDAC6. [file 1750-1172-8-66-S7.doc]

**Additional file 7**


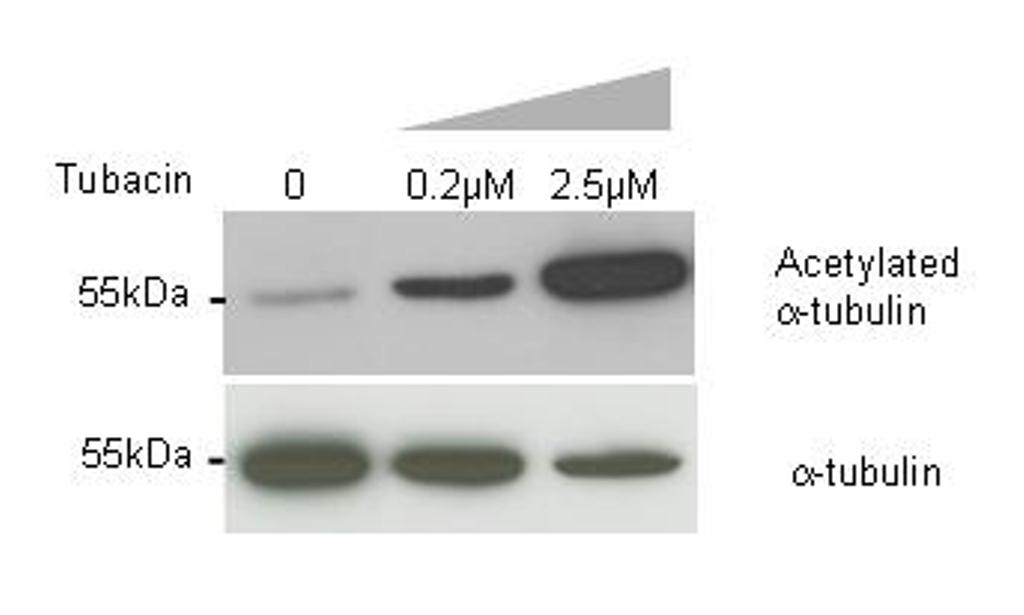


Western-blot analysis of control fibroblasts, using antibodies against acetylated α-tubulin (upper panel), and α-tubulin (lower panel), after treatment for 24h with a specific inhibitor of HDAC6, tubacin, at different concentrations (0, 0.2μM and 2.5 μM). Note that the α-tubulin antibody (Life Science) showed preferential immunoreactivity for deacetylated α-tubulin.
